# Sex differences in the molecular signature of the developing mouse hippocampus

**DOI:** 10.1186/s12864-017-3608-7

**Published:** 2017-03-16

**Authors:** Joseph L. Bundy, Cynthia Vied, Richard S. Nowakowski

**Affiliations:** 1 Department of Biomedical Sciences, Florida State University College of Medicine, 1115 West Call Street, Tallahassee, FL 32306 USA; 2 Translational Science Laboratory, Florida State University College of Medicine, 1115 West Call Street, Tallahassee, FL 32306 USA

**Keywords:** Hippocampus, Development, Transcriptomics, Proteomics, Sex differences

## Abstract

**Background:**

A variety of neurological disorders, including Alzheimer’s disease, Parkinson’s disease, major depressive disorder, dyslexia and autism, are differentially prevalent between females and males. To better understand the possible molecular basis for the sex-biased nature of neurological disorders, we used a developmental series of female and male mice at 1, 2, and 4 months of age to assess both mRNA and protein in the hippocampus with RNA-sequencing and mass-spectrometry, respectively.

**Results:**

The transcriptomic analysis identifies 2699 genes that are differentially expressed between animals of different ages. The bulk of these differentially expressed genes are changed in both sexes at one or more ages, but a total of 198 transcripts are differentially expressed between females and males at one or more ages. The number of transcripts that are differentially expressed between females and males is greater in adult animals than in younger animals. Additionally, we identify 69 transcripts that show complex and sex-specific patterns of temporal regulation through postnatal development, 8 of which are heat-shock proteins. We also find a modest correlation between levels of mRNA and protein in the mouse hippocampus (Rho = 0.53).

**Conclusion:**

This study adds to the substantial body of evidence for transcriptomic regulation in the hippocampus during postnatal development. Additionally, this analysis reveals sex differences in the transcriptome of the developing mouse hippocampus, and further clarifies the need to include both female and male mice in longitudinal studies involving molecular changes in the hippocampus.

**Electronic supplementary material:**

The online version of this article (doi:10.1186/s12864-017-3608-7) contains supplementary material, which is available to authorized users.

## Background

The formation and development of the mammalian brain involves neuronal proliferation and axonal growth followed by programmed cell death and pruning of synapses [1]. This tightly regulated process is governed by an ensemble of genes and signaling factors in both prenatal and early post-natal development. Transcriptional regulation of neural development has previously been explored using mouse models with high throughput technologies such as microarrays and RNA-sequencing (RNA-seq) [2–6]. These investigations have focused on a variety of brain regions, including the cerebral cortex [2, 3], hippocampus [6], and cerebellum [4]. One of these studies, an RNA-seq based investigation comparing cortical expression in the mouse embryo with that of animals aged 3–4 months, identified 4,125 transcripts changing expression from embryonic development into adulthood [2]. This finding reveals the substantial role of temporal regulation of the cortical transcriptome during neural development. However, these studies have either used one sex exclusively [2] or not included biological sex as a covariate of interest in data analysis [3–6].

Even prior to gonadal differentiation in utero, female and male brains have distinct patterns of gene expression as a result of chromosomal dosage differences [7, 8]. Additional sex differences in gene expression and brain morphology manifest as a result of the gonadal release of sex hormones both in utero and during postnatal sexual development [9–11]. These sex-specific gene expression signatures persist into adulthood in brains of both mice and humans [12, 13]. Our detailed investigation of sex differences in transcript expression in inbred mouse strains showed that molecular sex differences are influenced by genetic background, revealing tens to hundreds of differentially expressed (DE) transcripts depending on the strain [14], which might suggest that sex differences in humans are population-specific. Neural sex differences in mammals also manifest on the macroscopic scale. MRI and histological studies have identified sex differences in gross neuroanatomical features, such as cortical thickness in both mice and humans [9, 15].

Neural sex differences are also evident in human neurological disorders which have a sex-biased epidemiological profile. Males are more likely than females to be diagnosed with certain neurological disorders such as autism spectrum disorder [16, 17], dyslexia [18], Parkinson’s disease [19], and schizophrenia [20]. However, males are less likely to be diagnosed with Alzheimer’s disease [21] or major depressive disorder [22] than females of the same age. This is potentially concerning because many model-based molecular and behavioral investigations of these pathologies fail to include both female and male subjects [23]. Thus, important clues to disease cause and severity may be overlooked. The growing body of evidence for sex biases in both basic biology and clinical outcomes underscores the importance of understanding the differences between the molecular architecture of the female and male brain. Recently, this need has resulted in an NIH initiative that encourages the use of both female and male subjects in clinical trials and pre-clinical animal studies [24].

The hippocampus is an important telencephalic structure canonically associated with learning and memory, and is implicated in the pathology of several sex-biased neurological disorders. For example, Alzheimer’s Disease affects females more than males [21] and is associated with the loss of neurons in the hippocampus. Furthermore, patients with Major Depressive Disorder (MDD), a female-biased disorder, have reduced hippocampal volume relative to age and sex-matched controls [25]. In addition, the dentate gyrus of the hippocampus is one of only two sites of adult neurogenesis [26, 27], and hippocampal neurogenesis has been reported to be sexually dimorphic in young rats, with males rats producing more neurons with lower average survival rates than females [28]. Hippocampal spine synapse density is also sexually dimorphic in the mouse brain, and varies throughout the female estrous cycle [29, 30]. Taken together, these characteristics make the hippocampus a suitable candidate brain region for the investigation of sex-biased gene expression through development.

We have previously investigated sex differences in the hippocampal transcriptome in a variety of inbred strains [14] at a single age (60 days of age). Here, we expand on these findings by conducting a multi-omic analysis of the mouse hippocampus in postnatal development. In the current study, we document changes in the transcriptome and proteome of the female and male hippocampus in young (1 month old), pubescent (2 month old), and young adult (4 month old) mice using RNA-seq and liquid chromatography-mass spectrometry (LC-MS/MS) based proteomics. We identify: 1) genes that show changed mRNA expression during development, 2) transcripts that are differentially expressed between females and males at one or more stages of postnatal development, and 3) transcripts that have a sex-specific pattern of change through development.

## Methods

### Animals

Samples analyzed in this report are a subset of those used in a larger experiment focused on molecular pathology. Specifically, these samples are the sex- and age-matched controls in a study of sex differences in the 5XFAD mouse model of Alzheimer’s Disease [31], produced using the transgenic stock backcrossed into the C57BL/6J background (MMRRC stock number 034848-JAX). A 5XFAD colony is maintained by mating hemizygous 5XFAD transgenic mice (RRID: IMSR_JAX:006554; 5XFAD) to wild-type C57BL/6J mice (RRID: IMSR_JAX:000664; C57BL/6J) to produce 50% hemizygous 5XFAD mice and 50% non-transgenic wild-type controls. The non-transgenic wild-type progeny were used in this investigation of sex differences in non-transgenic mice on the C57BL/6J background. Adult female C57BL/6J and adult male 5XFAD mice were purchased from the Jackson Laboratory (Bar Harbor, ME) and housed in the Florida State University College of Medicine animal care facility. Animals were group housed and kept on a 12 h light-dark cycle. Food and water were provided ad libitum. All mice for this study were produced by mating hemizygous 5XFAD males to C57BL/6J females. The genotype of progeny was determined via standard PCR for the PSEN1 transgene including internal positive controls for PCR amplification consistent with the instructions provided by the Jackson Laboratory.

### Hippocampus dissection

Female and male mice of 1, 2, and 4 months of age (*n* = 5 for RNA-seq, *n* = 3 for LC-MS/MS) were decapitated and the hippocampus rapidly dissected as described in [14]. The brain was bisected and the diencephalon and brain stem removed so that the medial aspect of the telencephalon was accessible. The hippocampal formation was then “rolled out” and separated from the rest of the telencephalon. This dissection procedure produces a sample that contains the entire dorsal-to-ventral extent of the hippocampal formation with the dentate gyrus, CA3, CA2, CA1 and subiculum. The tissue break occurs at approximately the subiculum/presubiculum border. The hippocampal formation (from both sides of the brain) was then rapidly frozen in liquid nitrogen before being stored at -80°. Additional samples from the neocortex and cerebellum were also removed and frozen for future analysis. The total time between decapitation and deposition of the samples into the liquid nitrogen is ~2 min. All tissue samples used in this study were collected over the course of 2 months.

### Estrous staging

It has been shown previously that gene expression in the female mouse hippocampus is altered as a function of estrous stage [32]. Therefore, we determined the estrous stage of our female mice in order to eliminate estrous stage as a confounding factor. C57BL/6J mice begin cycling at approximately 60 days of age [33], therefore 2 month and 4 month (but not 1 month) animals were staged. A vaginal lavage was collected from females in the 2 and 4 month age groups immediately post-mortem. Vaginal smears were stained with crystal violet and the respective estrous stage of each mouse was determined via cell typological assessment as described in [34]. An insufficient number of mice were available to analyze tissue from mice in only one of the four stages of the estrous cycle exclusively. To ensure that the stage-to-stage variation was not a confounding variable across ages, we allocated the same number of samples in each estrous stage (two in estrus, two in metestrus, one in diestrus for RNA-sequencing, and one sample in proestrus, estrus, and metestrus for proteomics) for 2 and 4 month old females. All animal protocols were carried out in accordance with the AAALAC (Association for Assessment and Accreditation of Laboratory Animal Care) Guide for the Care and Use of Laboratory Animals: Eighth Edition and approved by the Institutional Care and Use Committee of Florida State University (Protocol # 1420).

### RNA extraction and CDNA library preparation

For the transcriptomic analysis, RNA from the dissected hippocampal tissue was extracted using a miRNeasy minikit (Qiagen, catalog #217004) with MaXtract high density columns (Qiagen, catalog #129056). A set of synthetic RNA standards were then added to 5 μg of RNA from each sample as spike-ins to assess the performance of each library (ERCC ExFold RNA Spike-In Mixes Ambion, catalog #4456739). mRNA was purified with a NEBNext mRNA magnetic isolation module (NEB, catalog #E7490L). cDNA libraries were generated from 50 ng of isolated mRNA using a NEBnext Ultra mRNA library preparation kit for Illumina sequencers (NEB, catalog #E7530L), and a unique 6-nucleotide index was incorporated to each library (NEB, catalog #E7335S, E7500S). The concentration of each cDNA library was estimated with qPCR (KAPA-PCR) using Illumina sequencing primers (KAPA Biosystems catalog # KK4835), and average fragment length was determined with a bioanalzyer high sensitivity DNA chip (Agilent Technologies, catalog #5076–4626). 12nM of each cDNA library was pooled into one of three cDNA library pools. Each of the three cDNA pools underwent additional quality control analysis via bioanalyzer and KAPA-PCR. 13pM of each cDNA library pool was sequenced on an Illumina HiSeq 2500 in the Translational Science Laboratory at the Florida State University College of Medicine.

Recent RNA-seq based transcriptomic investigations have demonstrated that sample-to-sample differences in library preparation and sequencing lane can confound quantitative assessments of RNA abundance and disallow comparisons of interest, particularly if all samples of one condition are prepared or sequenced in the same batch [35, 36]. Therefore, we took steps to reduce batch effects in sample handing and sequencing. All samples underwent various steps of processing (RNA extraction, mRNA isolation, cDNA library preparation) in sets of 6 in a semi-random fashion such that samples from each experimental group (i.e., all 2 month females) were not all co-prepared. Additionally, samples were non-randomly assigned to one of three cDNA library pools, each containing 20 cDNA libraries, such that each pool contained at least one library from each of the 6 experimental conditions. cDNA libraries were sequenced, with single end, 100 base reads on an Illumina HiSeq 2500 at the Florida State University College of Medicine Translational Science Laboratory.

### Protein extraction, isoelectric focusing, and LC-MS/MS

Protein extraction was performed via a modified FASP protocol [37] as described previously [38]. Briefly, hippocampal tissue from 1, 2, and 4 month old female and male mice (*n* = 3) was mechanically disrupted in extraction buffer with a mortar and pestle, sonicated, and boiled. Cellular debris was then removed via centrifugation, and the supernatant was serially washed in ultra-0.5 centrifugal filter devices (Amicon, catalog #UCF 501024) to remove mass-spectrometry incompatible reagents. Samples were quantified with a Qubit fluorometer (Life Technologies) and 200 μg of protein extract for each sample was digested with trypsin overnight. Digested lysates were isoelectrically focused into 12 fractions using an Agilent 3100 offgel fractionator. These 12 fractions were then pooled into 4 fractions to reduce experimental size and reduce analysis time. Subsequent to pooling, peptides were lyophilized and submitted to the Florida State University Translational Science Laboratory for (LC-MS/MS) analysis on a LTQ Orbitrap Velos high-resolution electrospray tandem mass spectrometer (Thermo Scientific) with the same instrument parameters described previously [38].

### Data analysis

#### RNA-sequencing

The RNA-seq data analysis workflow has been described previously [14]. Briefly, quality control analysis of each sequenced library was performed using the fastQC software. Removal of primer adapters, which were added as part of the library preparation protocol, was performed with Trimmomatic [39]. The trimmed sequencing reads were aligned and mapped using Tophat (v2.0.13) [40] to the mouse genome (genome release GRCm38) to assign each read to a gene. Following mapping with Tophat, reads were further processed (filtered, sorted and indexed) with Samtools [41] and only reads that mapped to a single gene were used for further analysis. Uniquely mapped reads were used to generate counts for each annotated gene using easyRNASeq [42]. A count table was generated for all samples containing the number of reads for each of the 37,315 annotated genes from the mouse genome. For differential expression analyses, RNA-seq data in the form of read counts were then analyzed with DESeq2 (version 1.8.1) [43]. For comparisons with LC-MS/MS proteomics data, Cufflinks (v2.2.1) [44] was used to generate FPKM (fragments per kilobase per million reads) values which are normalized for gene length and sequencing depth. The RNA-seq dataset supporting the conclusions of this article are available in the NCBI Gene Expression Omnibus [45] accession #GSE83931. Additionally, the read count table and metadata table are provided as Supporting Information (see Additional files 1 and 2).

#### Mass spectrometry

The LC-MS/MS data analysis workflow has been described previously [38]. Briefly, raw spectral data (.raw files) were uploaded into Proteome Discoverer (Thermo Scientific, version 1.4.0.288) using the MudPIT setting to combine data from multiple fractions corresponding to the same biological sample. Database searches were performed on each technical replicate with both Sequest HT and Mascot (version 2.4.0) using the target-reverse Mus musculus Swissprot reference proteome. Search result files in.msf format were then uploaded to the Scaffold software (Proteome Software, version 4.4.1.1). In Scaffold, the X!Tandem search option was selected. The following were used to select against spurious protein identifications in Scaffold: protein FDR = 1%, minimum # peptides = 2, peptide FDR = 1%. Spectral counts were normalized for protein length and sample loading with the NSAF option, and count data were exported from the Scaffold software. Ensembl gene IDs were downloaded for proteins detected in the experiment using the biomart tool on the ensemble website and added to data files. Spectral count data files were loaded into the R environment and matched with RNA-seq derived count data using ensemble gene IDs. The LC-MS/MS dataset supporting the conclusions of this article is available in the ProteomeXchange Consortium [46] via the PRIDE partner repository with the dataset identifier PXD004496. Additionally, spectral count data and LC-MS/MS sample metadata are provided as Supporting Information (see Additional files 3 and 4).

### Differential expression analysis

To identify transcripts and proteins that have changed expression between animals of different age or sex, we performed differential expression analyses with DESeq2 [47]. To identify transcripts that change in expression through all three ages (1, 2, and 4 months), we used the likelihood ratio test as implemented in DESeq2. We implemented this test using a full model with biological sex and animal age against a reduced model with biological sex as the only predictive variable, thus returning small *p*-values only for genes which have changed expression between animals of different ages. To investigate differences in transcript and protein expression levels between females and males, we performed pairwise comparisons of the sexes at each age using the exact test option as implemented in DeSeq2. Finally, to identify transcripts that both change over time and change *differently* in females than in males, we again used the likelihood ratio test from DeSeq2 using a full model with biological sex, animal age, and the interaction of biological sex and animal age. The reduced model for this test contained only biological sex and animal age predictive variables, thus returning small *p*-values only for genes which change over time in a sex-specific manner. For all differential expression analyses of RNA-seq data, the criterion for considering a transcript to be differentially expressed was an FDR-adjusted *p*-value (q) <0.05 [48].

### Enrichment analyses

Gene ontology (GO) and KEGG (Kyoto Encyclopedia of Genes and Genomes Expression Database, RRID: nif-0000-21234) enrichment analyses were used to aid in the interpretation of genes that are differentially regulated through development and between females and males. Enrichment analyses were conducted by uploading ENSEMBL gene identifiers to the WebGEstalt Toolkit [49, 50]. For both the GO and KEGG pathway enrichment analyses, the following criteria were used for filtering results: minimum number of genes per term = 5, p.adjust <0.05. All transcripts detected with at least one read count in the experiment were used as the “background” list for GO and KEGG enrichment. Lists of enriched GO terms were uploaded to the online tool REVIGO (REduce and VIsualize Gene Ontology) to create visualizations [51].

## Results

RNA sequencing was used to compare the transcriptional changes that occur in female and male C57BL/6J mice between 1, 2, and 4 months of age. Extracted mRNA was processed into cDNA libraries and sequenced on an Illumina HiSeq 2500, yielding a total of 441,688,394 reads across all 30 samples. Read counts uniquely mapped to a total of 25,520 transcripts, or 68% of the mouse genome (GRCm38).

**Fig. 1:**
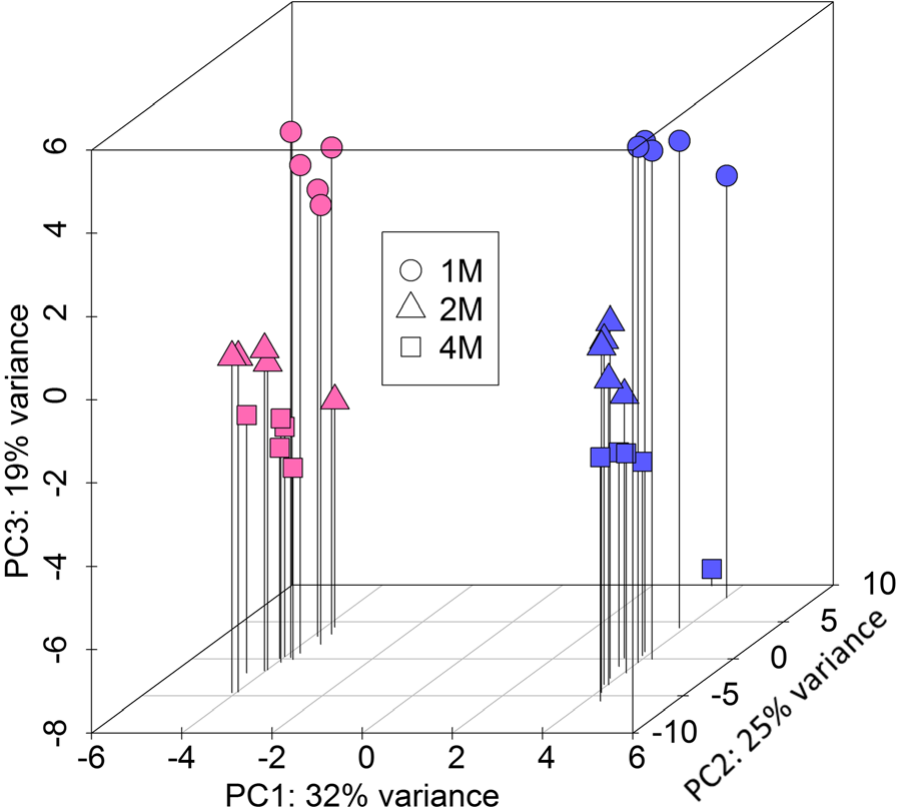
Principal component analysis. A 3D PCA plot was generated using normalized and variance stabilized transcript expression data (vst transformation, DESeq2) for the top 500 most variant transcripts in the dataset. The percent of variance explained by each principal comonent is displayed on each axis. Female samples (*pink*) and male samples (*blue*) cluster into two distinct groups on PC1. Within both the female and male clusters, samples of different ages (encoded by shape) cluster together on PC2 & PC3

To visualize gross gene expression differences between samples of different sex and age, we conducted a principal component analysis (PCA) with DESeq2. This analysis was based on transcript expression data in the form of variance stabilized read counts (DESeq2 VST transformation) for the 500 most variant transcripts in the dataset. PCA is a form of dimensional reduction that allows differences between samples to be visualized based on the major sources of variance in the data. The first three principal components contribute 76% of the total variance (32, 25 and 19%, respectively, and are shown in Fig. 1. The PCA shows a cluster structure that reflects differences in expression attributable to both age and sex (Fig. 1). Female and male samples at all 3 ages are segregated along the PC1 axis, indicating that the hippocampal transcriptome is distinct between the sexes. Within clusters of female and male samples, mice of different ages and both sexes are further segregated by contributions from both PC2 and PC3, confirming that the hippocampal transcriptome is altered through development in both females and males. Within the cluster of samples from the 4 month-old female mice, none of the principle components account for a substantial portion of the variance that might be attributed to the estrous cycle, and a scatter plot does not reveal any clustering by estrous stage (Additional file 5 Figure S1). This is consistent with a more detailed study in our laboratory (DiCarlo et al., manuscript in preparation).

### Temporal dynamics of the transcriptome in postnatal development

To identify the individual transcripts which 1) are differentially expressed at one or more ages, 2) are differentially expressed between the sexes, and 3) are differentially expressed over time differently between the sexes, we conducted a series of differential expression analyses using DESeq2.

**Fig. 2:**
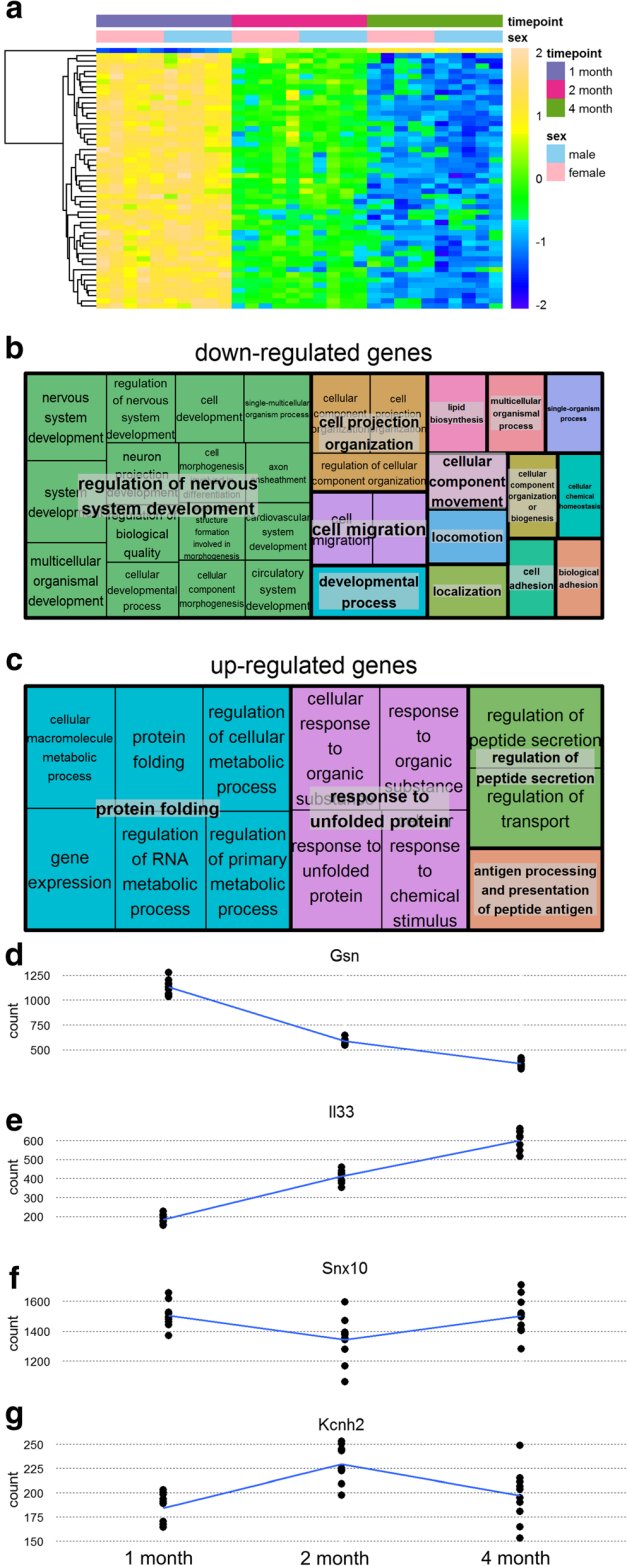
Changes in transcript expression through postnatal development. **a**: Heat-map of top 50 DE transcripts through development as ranked by *p*-value. Samples are organized into columns by age group, and transcripts are self-organized into rows by hierarchical clustering. Shading of boxes indicates relative levels of transcript expression within rows in terms of log2 fold change. *Tan* and *yellow* boxes indicate relatively high expression, and *blue* represents low expression. The majority of these transcripts [49] are down-regulated through the ages investigated, with only a single gene, *Il33* (*top row*), increasing in expression through postnatal development. **b**: GO enrichment treemap for transcripts that are down-regulated through development. Boxes represent biological processes disproportionately associated (enriched) with DE genes, with the area of boxes representing degree of enrichment for each term (as determined by FDR-adjusted *p*-value). Boxes of the same color represent similar gene ontology terms, grouped under a global GO term in bold. **c**: GO enrichment treemap for transcripts that are up-regulated through development. **d** - **g**: Example transcripts for each of the 4 patterns of temporal regulation observed. Normalized read counts are plotted on the y axis, and samples of different ages are plotted along the x axis. Lines are drawn through the mean count level at each age point

We first conducted a differential expression analysis to identify transcripts that change in expression as a function of age without differentiating between female and male samples. This analysis identifies 2699 transcripts that have altered expression through postnatal development. Numerous transcripts that have been previously associated with neural development appear in the top 50 DE transcripts (as assessed by *q*-value), such as: *Syt1* (synaptotagmin 1) [2, 3, 5, 6], *Gsn* (gelsolin) [2, 5], *Snca* (alpha-synuclein) [2, 3, 5], *Mbp* (myelin basic protein) [2], and *Dcx* (doublecortin) [2] Almost all of the top 50 DE transcripts show a monotonic down-regulated pattern (Fig. 2a). The single exception is *Il33* which has a monotonic up-regulated pattern (Fig. 2a and e). For the total population of 2699 DE genes, there are 4 patterns of age-related transcript expression changes; examples of each of the 4 patterns of expression are shown in Fig. 2d-g. The majority (63%) of temporally regulated transcripts display monotonic changes in expression (i.e., expression levels that only increase or decrease over time). Of these, 949 (35%) transcripts show progressive down-regulation at 2 and 4 months of age from an observed maximum level of expression at 1 month (Fig. 2a and d). A slightly smaller proportion, 752 (28%), of the transcripts display a consistent pattern of up-regulation through postnatal development to peak expression at 4 months of age (Fig. 2a and e). Of the 37% of DE transcripts with more complex patterns of regulation, 551 (20%) transcripts decrease in expression from 1 to 2 months of age, and are subsequently up-regulated from 2 to 4 months of age (Fig. 2f). Four hundred forty seven (17%) transcripts display the inverse pattern of expression, and are up-regulated from 1 to 2 months and subsequently down-regulated from 2 to 4 months of age (Fig. 2g).

We conducted GO enrichment analyses on subsets of differentially expressed transcripts to identify biological processes associated with changes in expression. Down-regulated transcripts show enrichment for terms such as “nervous system development”, “neurogenesis”, “myelin sheath” and “neuron projection development” (Fig. 2b). This is expected, as postnatal development and maturation is a period marked by extensive neurological change, including an increase in myelination, a decrease in neuronal proliferation, and the pruning of synapses [1]. However, also enriched in the dataset are GO terms such as “lipid biosynthesis”, which are less obviously associated with development. KEGG pathway analysis shows enrichment for processes that are associated with neural development such as “Axon guidance”. Surprisingly, several immune system associated terms are also present, such as “Bacterial invasion of epithelial cells” and “Leukocyte transendothelial migration”. It is known that hundreds of immune system associated transcripts increase in expression throughout the brain through the normal aging process [52]. However, it is unclear what roles these immune system associated genes play in postnatal development. Interestingly, GO analysis of transcripts up-regulated through postnatal development returns a set of enriched GO terms, most of which are associated with “protein folding” (Fig. 2c).

### Repression of gene expression in postnatal development

**Fig. 3:**
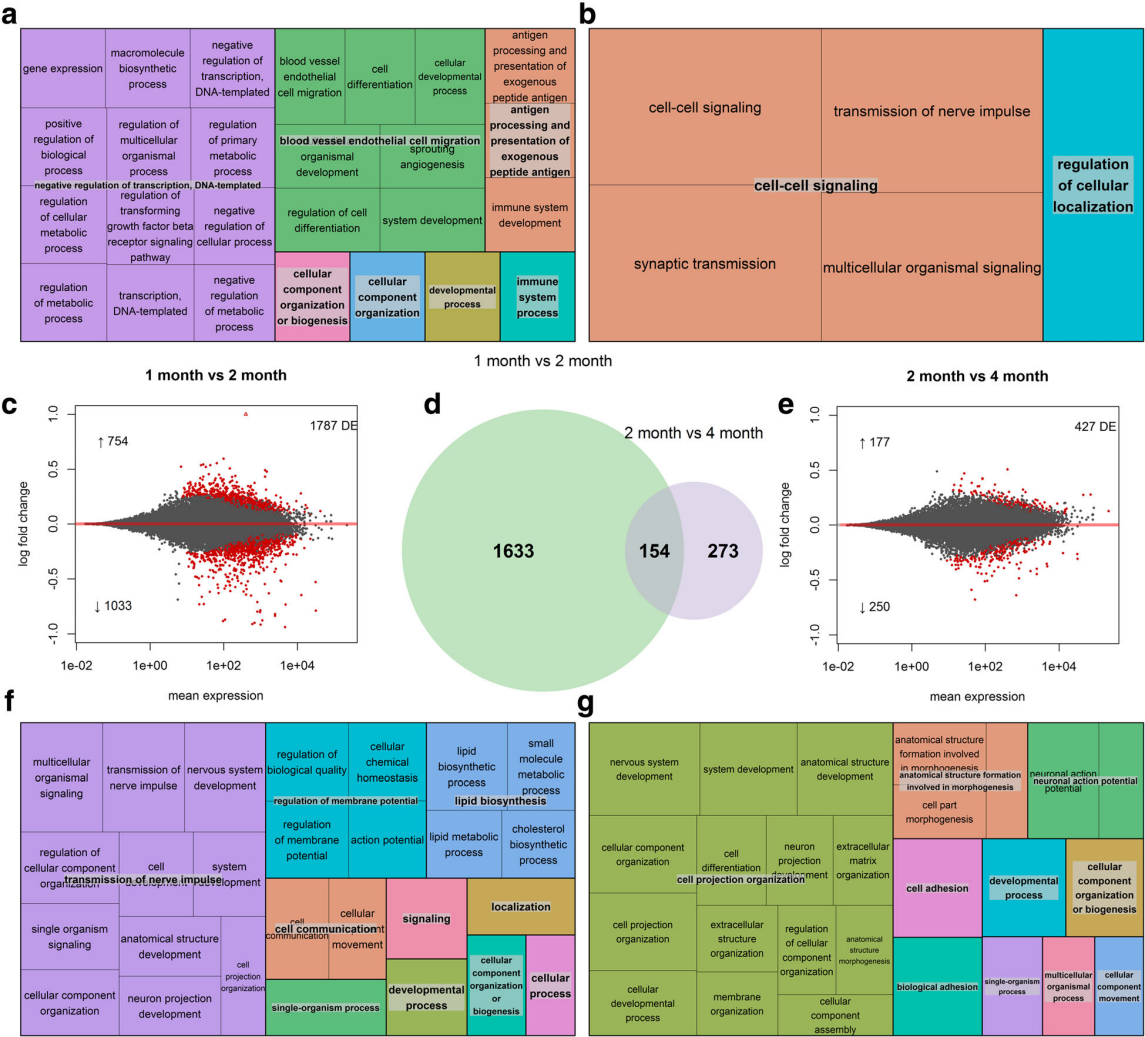
Changes in expression through discrete periods of postnatal development. **a & b**: GO enrichment treemap for transcripts upregulated between 1 and 2 months, and 2 and 4 months respectively. **c**: shotgun plot for comparison of 1 and 2 month old animals. The log transformed fold change for each gene is plotted along the y axis, and the mean read count value is plotted along the x axis. Log2 fold changes were calculated by taking the log (base2) of the quotient of mean counts in relatively older animals (numerator) and relatively younger animals (denominator). Transcripts with more numerous read counts appear further to the right. *Red* points are genes that are up or down-regulated significantly (q < 0.05). **d**: Venn diagram comparing genes DE between 1 and 2 month, and 2 and 4 months of age. **e**: shotgun plot for comparison of 2 and 4 month old animals. 3**f** & 3**g**): GO enrichment treemap for transcripts down-regulated between 1 and 2 month and 2 and 4 months respectively. For c and e the number of total differentially expressed transcripts is shown in the upper right corner and the number of up-regulated vs down-regulated transcripts is shown with an up or down arrow

To better determine the periods in postnatal development in which genes change expression, we conducted pairwise comparisons of 1 vs 2 month old animals and also of 2 vs 4 month old animals (Fig. 3). One thousand seven hundred eighty seven transcripts change expression between 1 and 2 months of age, 1033 (58%) of which are down-regulated (Fig. 3c). As expected, many GO terms canonically associated with neural development are enriched in this gene set, such as “neurogenesis”, “neuron projection development”, and “neuron spine” (Fig. 3a and f). The KEGG enrichment analysis also identified several significantly enriched pathways associated with neural development, such as “Axon guidance” and “Focal adhesion”. Several immune system associated pathways also show significant enrichment, such as “Bacterial invasion of epithelial cells”, “Leukocyte transendothelial migration”, and “Chemokine signaling pathway”. Interestingly, *Il33*, a gene not previously implicated in hippocampal development is the most substantially up-regulated transcript, increasing expression by 125% between 1 and 2 months of age (Fig. 2e).

Comparisons of 2 and 4 month old animals reveal that fewer transcripts (427) change expression in later periods of postnatal development (Fig. 3e). Again, more transcripts show down-regulation (250) than up-regulation (177). Only 154 transcripts that show expression changes between 2 and 4 months of age also have altered expression between 1 and 2 months (Fig. 3d). Among these transcripts is *Il33*, again ranking as the most substantially up-regulated gene, increasing an additional 46% from 2 to 4 months (for a total of 229% at 4 months from its lowest expression at 1 month of age). Despite the relatively modest overlap in gene sets, the differentially expressed transcripts between 2 and 4 months of age show enrichment for many GO terms associated with the same neurodevelopmental processes enriched in the 1 vs 2 month comparison, such as “neuron projection development” and “transmission of nerve impulse” (Fig. 3a, b, f and g).

### Increase in sex-biased gene expression through postnatal development

**Fig. 4:**
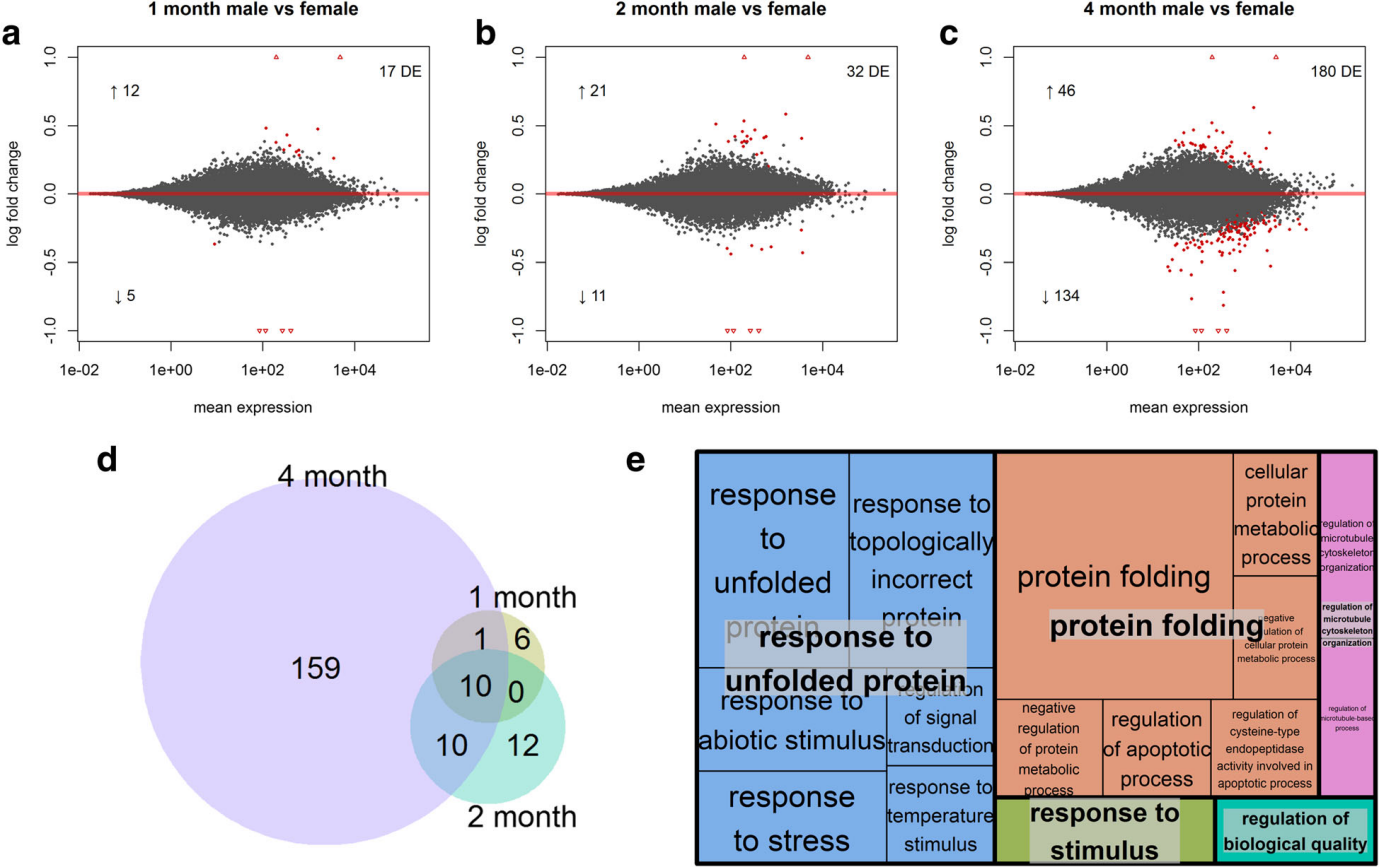
Sex biased gene expession through development. **a** - **c**: Shotgun plots for comparisons of females and males at 1, 2, and 4 months of age. In each plot the number of total differentially expressed genes is shown in the upper right corner and the number of up-regulated vs down-regulated transcripts is shown with an up or down arrow. Log2 fold changes were calculated by taking the log (base2) of the quotient of mean counts in female animals (numerator) and male animals (denominator). **d**: Venn diagram of sex-biased transcript expression at 1, 2, and 4 months of age. **e** GO enrichment treemap for transcripts with sex-biased expression at 4 months of age

To identify transcripts with different levels of expression between the sexes, we performed pairwise comparisons of age-matched females and males using the exact test option in DESeq2 (Fig. 4). Sex-biased expression is found for 17, 32 and 180 genes in 1, 2, and 4 month old animals, respectively (Fig. 4a, b and c). The increase in sex-biased transcript expression before, during, and after sexual development is consistent with the hypothesis that the release of sex hormones at approximately 2 months of age drives many sex differences observed in adult mice. Surprisingly, the set of DE transcripts in early age points are not subsets of DE transcripts in later age points (Fig. 4d). At 1 and 2 months of age, 6 and 12 transcripts have sex-biased expression, unique to each age respectively. However, the expression profiles of these 18 transcripts reveal patterns of expression reflecting possible sex-biased differences in other ages, but do not achieve significance in all comparisons. In 1 and 2 month old animals, most sex-biased transcripts are more highly expressed in females (female-biased) than in males. This is expected, as several of these transcripts (i.e., *Kdm6a*, *Ddx3x*, and *Eif2s3x*) are products of X-linked genes that escape X-inactivation, and have been shown to have sex-biased expression in the hippocampus [53, 54]. A substantial proportion (29%) of transcripts showing sex-biased expression in young (1 and 2 month old) animals are sex-linked (on X or Y chromosomes). Thus, much of sexually dimorphic transcript expression at these ages is likely driven by dosage differences of the sex chromosomes. In contrast to the preponderance of female-biased expression in 1 and 2 month old animals, almost three times as many transcripts (134 vs 46) are male-biased than female-biased in comparisons of 4 month old animals (Fig. 4c). Additionally, the majority (92%) of these genes are located on autosomes, 73% of which have male-biased expression. Interestingly, transcripts that show sex-biased expression at 4 months of age, which are primarily overexpressed in males, show GO enrichment for several terms associated with “protein folding” and “regulation of apoptotic process” (Fig. 4e).

### Sex-specific transcriptional regulation through development

**Fig. 5:**
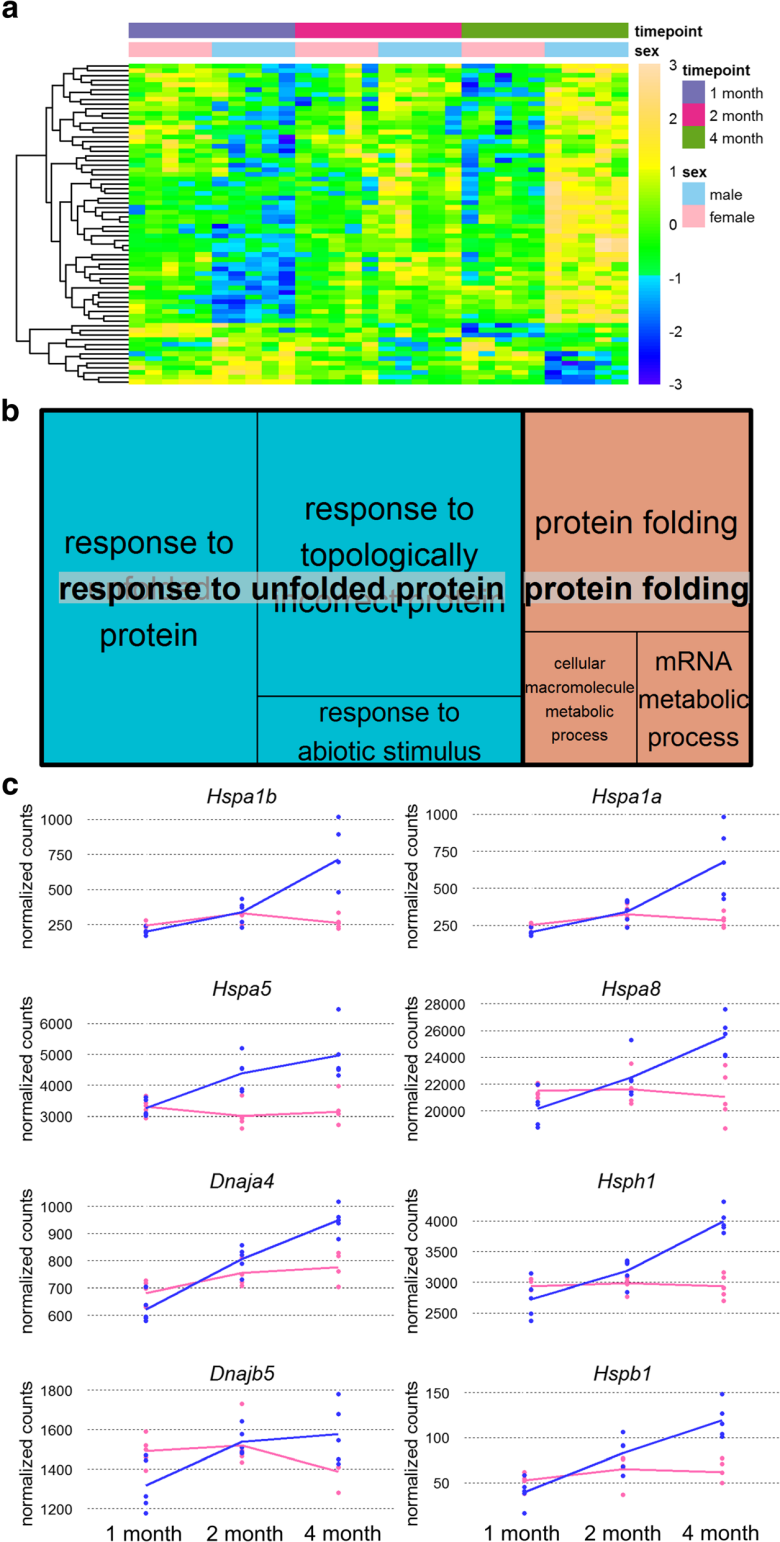
Sex-specific patterns of temporal regulation. **a**: heatmap for the complete set of 68 transcripts that show significant sex-specific patterns of change in expression at one or more ages. Samples (columns) are arranged by age group, with females and males also grouped together within age groups. Relative expression values for individual genes are organized into rows. Relative expression values for each gene were calculated by dividing the normalized count value for each sample by the mean of count values for that gene across all samples. The result was then log2 transformed and used for shading. The majority of these transcripts are up-regulated through development in males relative to females. **b**: GO enrichment treemap for genes shown in heatmap. **c**: Transcript expression profiles for the 8 heat-shock proteins found to change over time in a sex-specific manner in this comparison. Lines are drawn through the mean count level at each age point for both females and males in *pink* and *blue*, respectively

To identify transcripts that 1) change over time and 2) show a different pattern of change in females and males, a likelihood ratio test was performed using DESeq2. This analysis identifies 68 transcripts that change over time in a sexually dimorphic manner (Fig. 5a). GO analysis of this transcript set shows enrichment for the GO terms “protein folding” and “response to misfolded protein” (Fig. 5b). KEGG analysis of these genes returns a single term, “Protein processing in endoplasmic reticulum”. The primary source of the enrichment for these terms is 8 heat shock proteins (HSPs) that display a sexually dimorphic pattern of expression change in the mouse hippocampus through development (Fig. 5c). These transcripts have similar to slightly higher expression in females relative to males at 1 month of age. At 2 months of age, the relative difference in gene expression is reversed, with transcript abundance higher in males. This difference in expression between the sexes increases at 4 months of age for all of the 8 HSPs, with male transcript abundance an average of 70% higher than that of females.

**Fig. 6:**
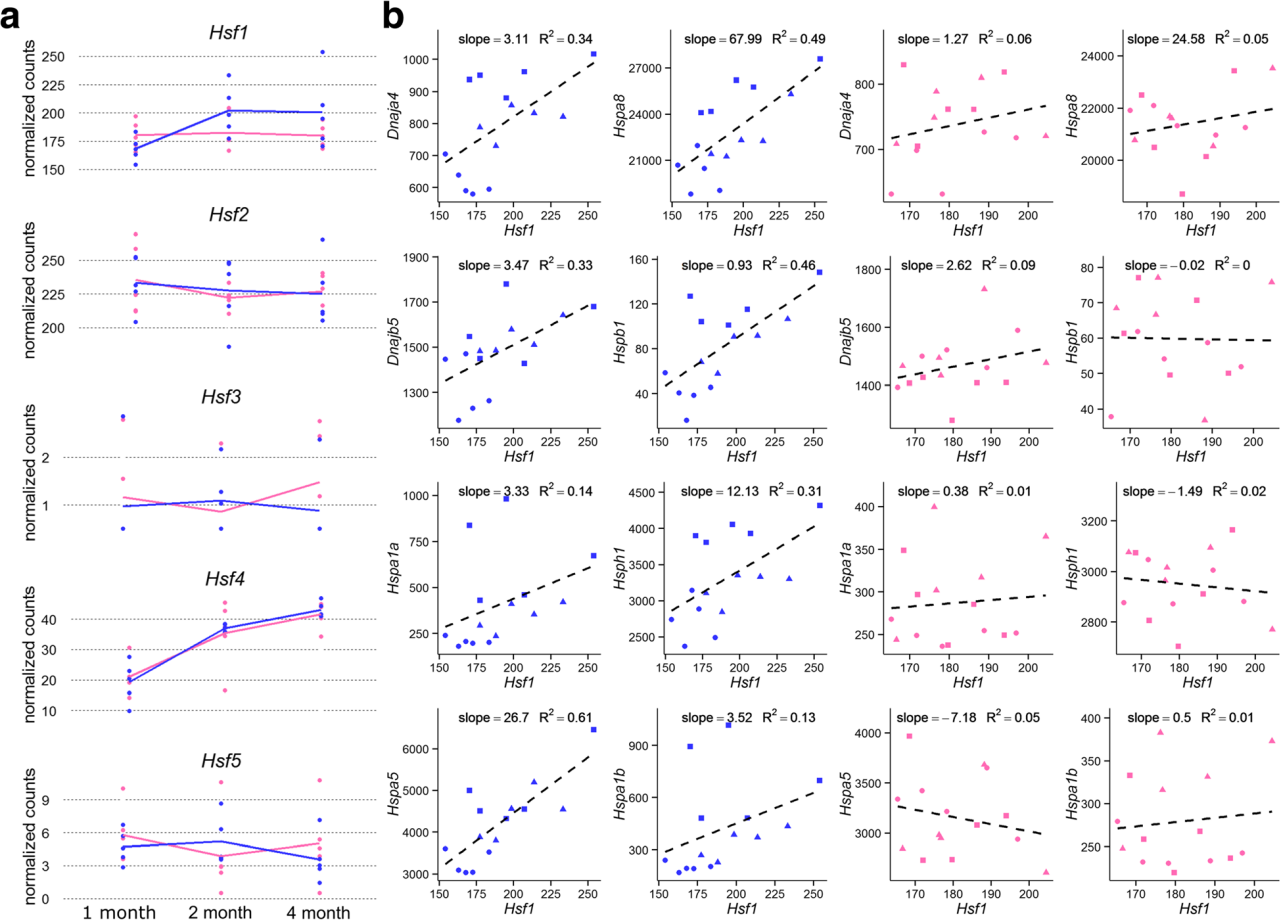
Expression profiles and linear regressions of heat shock factors. **a**: Gene expression profiles for *Hsf1-5*. Female and male samples and trendlines (through sample means) are shown in *pink* and *blue*, respectively. **b** Linear regression analyses of *Hsf1* and HSP mRNA levels in female (*pink*) and male (*blue*) samples. One, 2, and 4 month old animals appear as circles, triangles, and squares, respectively. Normalized read counts for *Hsf1* are plotted along the x axis, and normalized read counts for each HSP are plotted on the y axis

**Fig. 7:**
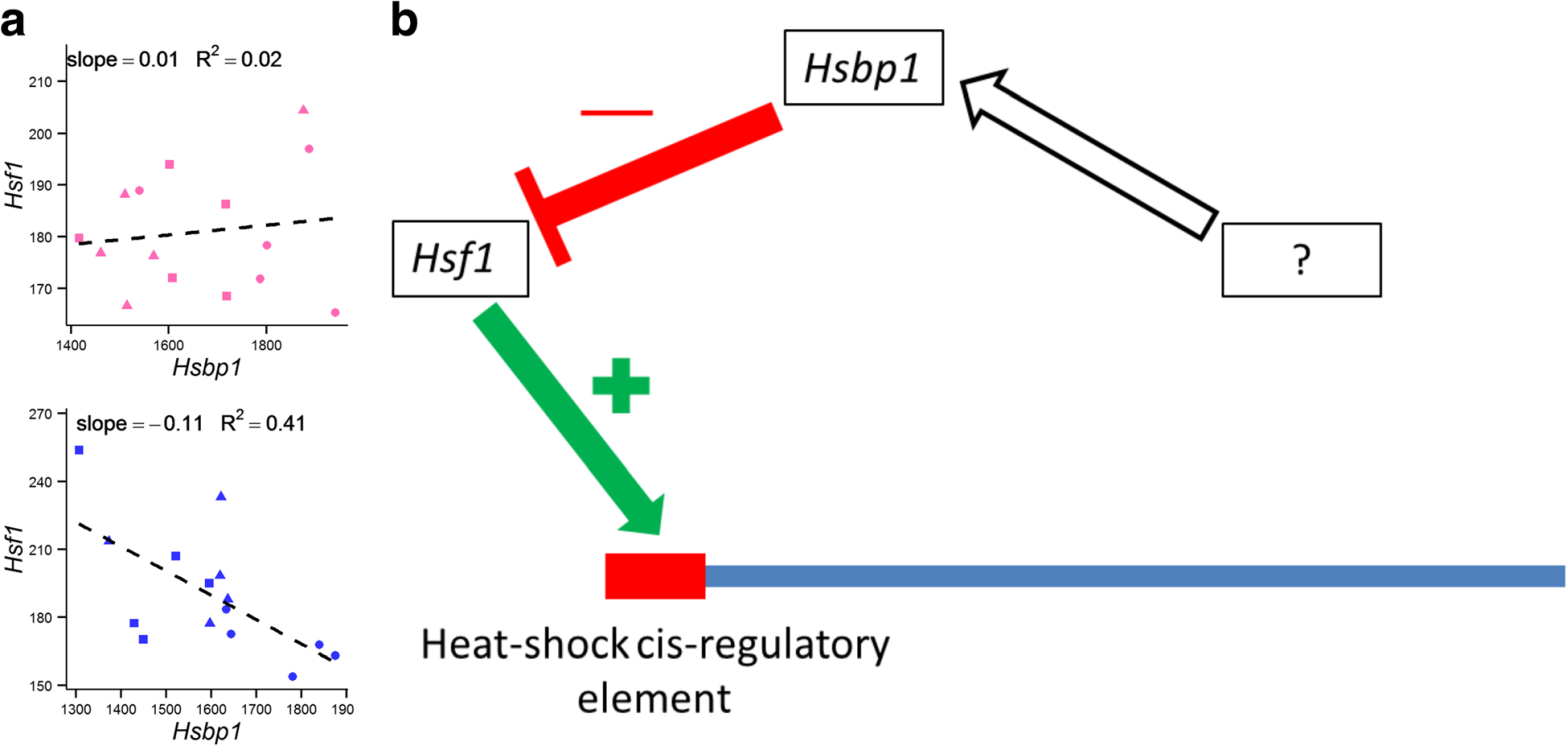
Sex-specific regulation of HSPs by *Hsf1* and *Hsbp1*. **a**: Linear regression analysis of *Hsbp1* vs *Hsf1* levels in females (*pink*) and males (*blue*). One, 2, and 4 month old animals appear as circles, triangles, and squares, respectively. **b**: Speculative model for sex-specific induction of HSP expression

In an attempt to elucidate the factors regulating HSP expression in our data, we investigated the transcript expression patterns of genes known to regulate HSP expression. HSPs are transcriptionally induced by a family of heat-shock specific transcription factors (HSF) [55]. The transcript expression profiles for these transcription factors are shown in Fig. 6a. Of the 5 transcription factors in this family, only *Hsf1* displays a pattern of expression similar to that of the 8 DE HSPs, suggesting that *Hsf1* plays a role in mediating the sex-difference in HSP expression. To better determine if *Hsf1* is implicated in regulating HSPs in a sexually dimorphic manner, we directly explored the relationship between the levels of the 8 HSPs and *Hsf1* by linear regression (Figs. 6b). Interestingly, levels of *Hsf1* are significantly correlated with expression of the 8 HSPs in males but not in females (cor.test in R, *p* < 0.05). We then investigated the expression patterns of factors upstream of *Hsf1* to determine if they are implicated in driving sex-biased HSP expression. *Hsf1* expression is known to be transcriptionally repressed by *Hsbp1* (heat shock binding protein 1). *Hsbp1* binds to the oligomeric domain of *Hsf1* and impedes DNA-binding, thus repressing both *Hsf1* and HSP transcription [56]. A regression analysis of *Hsbp1* and *Hsf1* levels shows a significant and negative relationship between *Hsbp1* and *Hsf1* in males but not females (Fig. 7a). These data suggest that both *Hsf1* and *Hsbp1* likely play a role in mediating sex-biased expression of HSPs in the mouse hippocampus. However, it remains unclear what factors upstream of *Hsbp1* and/or *Hsf1* regulate their expression in a sex-biased manner (Fig. 7b).

### Transcriptome - proteome comparisons

**Fig. 8:**
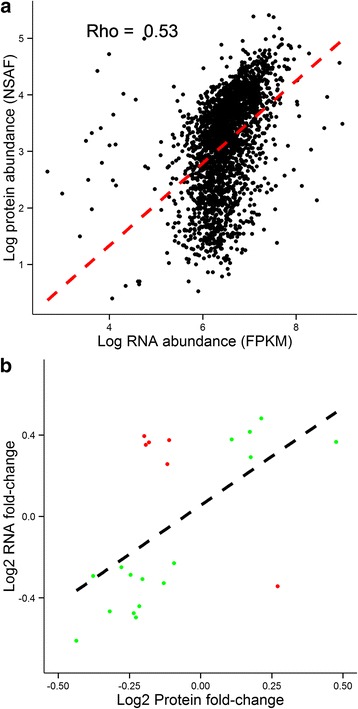
Comparisons of mRNA and protein abundances. **a**: Linear regression analysis for mean mRNA abundance (FPKM) and protein abudance (NSAF) across all samples reveals a stasticially significant (cor.test in R, *p* < 0.05) though modest correlation between mRNA and protein as measured by Spearman rank correlation coefficient (Rho). Log2 transformed RNA abundance values (FPKM) are plotted along the x axis, and log2 transformed protein abundance values are plotted along the y axis. **b**: Linear regression analyis for mRNA and protein fold changes for comparison of 1 and 2 month old animals. Only genes with changed mRNA (*q*-value < 0.05) and protein (*p* value <0.05) are shown. The majority of these genes (16 of 22) have mRNA and proteins that change in the same direction from 1 to 2 months of age (green points). Six of the 22 genes have a protein log2 fold change that has a different sign than the RNA log2 fold change (*red* points)

In addition to an RNA-seq based interrogation of the transcriptome, we also used mass spectrometry to identify proteins with changed expression in the hippocampus of females and males during the same periods of postnatal development. In total, 2540 proteins were identified with at least two spectral counts excluding common contaminants (see methods for filtering criteria). Pairwise comparisons between animals of different age and sex identified no proteins with significantly different abundance (q < 0.05), likely due to the relatively low number of biological replicates in these comparisons (*n* = 3). Proteins were matched with their corresponding mRNAs using a common gene ID provided by the online biomart tool by ensemble (ensembl.org/biomart). Of the 2540 gene products present in the LC-MS/MS dataset, 2517 are also present in the mRNA dataset. A regression analysis of the mean mRNA and protein abundance across all samples reveals a statistically significant (cor.test in R, *p* < 0.05) though modest correlation (spearman rank coefficient = 0.53) between the transcriptome and proteome (Fig. 8a). This is consistent with previous investigations of mRNA and protein correlations in complex samples, which report spearman correlation coefficients between 0.45 and 0.74 [57]. Focusing on comparisons of different age groups in which female and male samples could be pooled into a single category (all 1 month old animals vs all 2 month old animals, where *n* = 6), we investigated how well changes in protein abundance were predicted by changes in RNA abundance. We focused our analysis further by considering only the subset of genes which showed both significant differences in mRNA levels, and a trend towards changed protein abundance through development. Of the 2517 genes for which both mRNA and protein product were detected, only 22 genes (Table 1) show changes in both mRNA and protein levels (*q*-value <0.05 for RNA, *p*-value <0.05 for protein). To see how changes in mRNA levels correlate with changes in protein abundance of these 22 genes, we performed regression analyses of protein fold change between 1 and 2 month old animals against RNA fold change (Fig. 8b). The majority of proteins change expression over time in the same direction as their respective RNA (16 genes, Fig. 8b, green points), confirming that transcriptional modulation of gene expression is an important determinant of protein expression in the hippocampus. Interestingly, a minority of genes (6 genes, Fig. 8b, red points) change in the opposite direction of the respective mRNA fold change. Among these genes are *Syn2* (Synapsin II) and *Sv2b* (synaptic vesicle glycoprotein 2B), both of which are known to modulate neurotransmitter release [58, 59]. Both of these genes show significant down-regulation of mRNA (*q*-value <0.05) from 1 to 2 months of age, but up-regulated protein expression (*p*-value <0.05). These observations indicate that post-transcriptional forms of regulation also play a role in regulating hippocampal gene expression through postnatal development.

**Table 1:** Log2 fold changes and significance staistics for genes with significantly changed mRNA and protein expression between 1 and 2 months of age

Gene Symbol	RNA FC	RNA *q* value	Protein FC	Protein *p* value
Qdpr	0.48	1.29E-23	0.36	5.77E-03
Car2	0.21	2.26E-04	0.48	2.32E-04
Pfkl	0.18	1.79E-02	0.29	1.97E-02
Plec	0.17	7.10E-04	0.42	4.43E-05
Cplx2	0.11	3.66E-02	0.38	1.95E-02
Actg1	−0.09	3.42E-02	−0.23	1.46E-02
Rdx	−0.13	2.08E-02	−0.33	3.37E-02
Prkar2b	−0.20	8.65E-03	−0.31	3.33E-02
Mapre1	−0.22	7.35E-07	−0.44	1.13E-02
Oxct1	−0.23	8.32E-11	−0.50	2.34E-06
Dpysl4	−0.23	5.68E-06	−0.48	5.25E-04
Tubb2b	−0.25	7.73E-07	−0.29	4.33E-03
Tuba1a	−0.28	2.83E-11	−0.25	4.26E-03
Strn	−0.32	7.05E-09	−0.47	1.11E-02
Dpysl5	−0.38	4.20E-10	−0.29	2.09E-02
Dpysl3	−0.44	2.25E-25	−0.61	1.10E-07
Acaa2	0.27	1.67E-02	−0.34	1.90E-02
Blmh	−0.11	3.74E-02	0.37	4.08E-02
Syn2	−0.12	1.69E-02	0.26	1.70E-02
Cd81	−0.18	2.77E-08	0.36	1.75E-02
Sv2b	−0.19	1.19E-04	0.35	1.60E-02
Rhog	−0.20	1.78E-02	0.39	2.37E-02

## Discussion

This investigation centers on changes in the transcriptome and proteome in the hippocampus as a function of two biological variables: the stage of postnatal development, and biological sex. We build on previous investigations by identifying transcripts that are not only temporally regulated, but that also change through postnatal development in a sexually dimorphic manner. Our analysis indicates that 2699 transcripts change significantly in the hippocampus between 1, 2, and 4 months of age, many of which are associated with known neurodevelopmental processes such as “cell morphogenesis” and “cell projection organization”. Among these transcripts are many genes already shown to have changed expression in other brain regions during periods of development, (i.e., *Gsn*, *Mbp*) [2, 5]. In supplement to previous findings, we also identify several transcripts which are not known to undergo significant regulation in postnatal development, such as *Il33*.

Our analysis indicates that most transcripts that display monotonic changes in expression across age points are down-regulated. However there is also a subset of genes that are up-regulated substantially. The significant enrichment of immune system associated GO terms in the comparison of 1 month and 2 month old animals indicates that several immune system associated genes play a role in neural development. Among these is *Il33*, a known pro-inflammatory cytokine, which displays a profound increase in expression through postnatal development. Indeed, *Il33* is the most highly up-regulated transcript through postnatal development in the hippocampus (Figs. 2e, c and e). *Il33* is known to be a potent regulator of lymphocyte recruitment. It has also been shown to bind heterochromatin and repress transcription in vivo [60]. Our data suggest that *Il33* may play an as yet unrecognized role in down-regulating gene expression in the mouse hippocampus through early postnatal development.

Unlike *Il33*, a subset of the genes (i.e., *Snx10*, *Kcnh2*) display non-monotonic pattern of expression (Fig. 2f and g). We emphasize this minority of genes, as it provides context for the observation that several transcripts found to have changed expression through the ages investigated show the opposite trend in our data relative to that reported in previous investigations of neural development. For example, *Mobp* is up-regulated by almost 9000-fold from embryonic day 17 to postnatal day 60 in the cerebral cortex [2]. However, in our dataset, *Mobp* is one of the most down-regulated transcripts through the postnatal development stages that we have analyzed, and decreases by 38% between 1 (P30) and 2 months (P60), and an additional 17% between 2 (P60) and 4 months (P120) of age. Several myelin-associated transcripts, such as *Mobp* and *Mbp*, are up-regulated throughout the brain from E18 to P4 [61]. Therefore, the disparity in these observed patterns of gene expression is likely due to the unique set of developmental periods investigated in our study relative to other investigations, as opposed to differences in transcriptional regulation across brain regions. Our data demonstrate that many genes do not increase or decrease uniformly through postnatal development. Therefore, different studies focusing on different sets of developmental periods (such as embryonic day 17 and postnatal day 60 in [2], and P30, P60, and P120 in our study) are likely to reach different conclusions regarding whether genes are up or down-regulated. In early periods of postnatal development, these transcripts are upregulated to provide the molecular constituents of the myelin sheath to enable salutatory conduction. However, our data demonstrate that these myelin-associated genes are subsequently down-regulated in the hippocampus between 1 and 4 months of age, coincident with a period of neural development marked by substantial synapse elimination and axonal pruning. Thus, our data emphasize that mammalian brain development is a complex, multi-phased process associated with wide-spread regulation on the transcriptional level, and that genes that are up or down-regulated at one developmental stage may not retain that pattern of expression in the next.

Most importantly, this study shows that layered on top of the broad changes in gene expression that take place in postnatal development, the transcriptional signatures of the developing female and male hippocampus are distinct. The number of transcripts showing sex biased expression increases from 17, to 32, to 180 at 1, 2, and 4 months of age, respectively. Interestingly, the relatively small number of sex-biased genes in 4 month old animals show high enrichment of a biological process not typically associated with sex differences in neural gene expression: “protein folding”. This biological process is enriched in both differential expression analyses focused on sex differences: 1) the pairwise comparison between 4 month old females and males (Fig. 4c) and, 2) The likelihood ratio test identifying transcripts with sex-specific temporal regulation (Fig. 5b). In both sets of DE genes, the transcripts which drive the enrichment of “protein folding” are HSPs (Fig. 5c). These transcripts show stable expression in females across the ages we investigated, but are progressively up-regulated in males, achieving a level that is significantly different from females at 4 months of age. A subset of these differences is also recovered in the LC-MS/MS dataset. Of the 8 HSP transcripts that are differentially regulated between the sexes, 4 were quantified by LC-MS/MS, 3 of which were higher (though not significantly) in males than females, the fourth showing very little difference between the sexes (Table 1).

Heat shock proteins were originally characterized in *Drosophila bushii* by their increased expression in response to increased temperature [62]. These proteins are known to function as chaperones, promoting the folding of proteins into biologically active states and preventing aberrant folding [63]. The induced expression of several HSPs (such as *Hsp70*) has been shown to promote the survival of mammalian cells when exposed to heat stress [64]. HSP expression is regulated by a family of transcription factors (*Hsf1-5*) which in response to various stimuli, bind the heat shock element and induce transcription [55]. Of these five factors, only *Hsf1* shows a pattern of sexually dimorphic change in expression through development correlated with that of the differentially expressed heat shock proteins in our data (Fig. 6a). These data suggest that *Hsf1* may play a role in inducing HSP expression in the male hippocampus in development, and that HSP expression in the female hippocampus may be less sensitive to induction by *Hsf1*. To our knowledge, heat-shock proteins are not known to play a substantial role in postnatal hippocampal development, nor are they known to have a sex-biased signature of expression. However, previous investigations using knockout mice show that HSPs may have a role in maintaining dendritic spines [65]. That these chaperones are up-regulated only in males at adulthood presents a candidate mechanism by which excessive protein aggregation could by attenuated in males but not females. If this pattern of sex-biased expression of heat-shock proteins is conserved across mammals, these data may present a candidate molecular correlate for the sex bias observed in Alzheimer’s disease, a disease characterized by aggregation of amyloid beta in neurons which is more prevalent in women than men. Future studies focusing on the levels of HSPs in human females and males would be helpful in determining whether: 1) there is a sex difference in HSP expression in the human hippocampus and 2) increased HSP expression is associated with attenuated AD development or progression.

## Conclusions

To expand on previous studies of molecular correlates of neural development and sex differences, we performed a transcriptomic investigation of mRNA levels in the developing mouse hippocampus before, during, and after sexual development. This analysis identifies a preponderance of transcriptional regulation through postnatal development, consistent with the findings of previous investigations. Additionally, we identify a host of transcripts that show sex-specific regulation through development, even prior to sexual development. Several of these transcripts are heat-shock proteins, which have previously been shown to promote cell survival, present a possible molecular basis for sex biases in neurodegenerative disorders. These findings clarify the need to consider sex differences in studies that investigate hippocampal development, even in periods prior to sexual maturation. Planned future investigations include targeted molecular investigations of heat shock protein levels in females and males at later time points, as well as the investigation of splice variation through development in the current RNA-seq dataset.

## Additional files

Additional file 1: Read count tables for RNA-seq assay. File contains quantitative information from transcriptomic investigation of gene expression in the hippocampus in both raw (unnormalized) and normalized (FPKM) formats. (XLSX 15052 kb)
Additional file 2: Metadata table for RNA-seq assay. File contains information on RNA-seq samples such as sample ID, sex, timepoint, and estrous stage. (CSV 749 bytes)
Additional file 3: Spectral count tables for LC-MS/MS assay. File contains quantitative information from proteomic investigation of protein expression in the hippocampus in both raw (unnormalized) and normalized (NSAF) formats. (XLSX 682 kb)
Additional file 4: Metadata table for LC-MS/MS assay. File contains information on LC-MS/MS samples such as sampleID, sex, timepoint, and estrous stage. (CSV 622 bytes)
Additional file 5: Figure S1. Principal Component Analysis of only female samples. File contains figure of principal component analysis of 2 and 4 month old female samples to investigate variance in gene expression associated with differences in estrous stage. (PNG 96 kb)

## Abbreviations

DE: Differentially expressed
GO: Gene Ontology
HSP: Heat-shock protein
