# Supplementary figures and images for: Sex differences in the molecular signature of the developing mouse hippocampus
Source: BMC Genomics. 2017 Mar 16;18:237. doi: 10.1186/s12864-017-3608-7 (PMC5356301; doi:10.1186/s12864-017-3608-7)

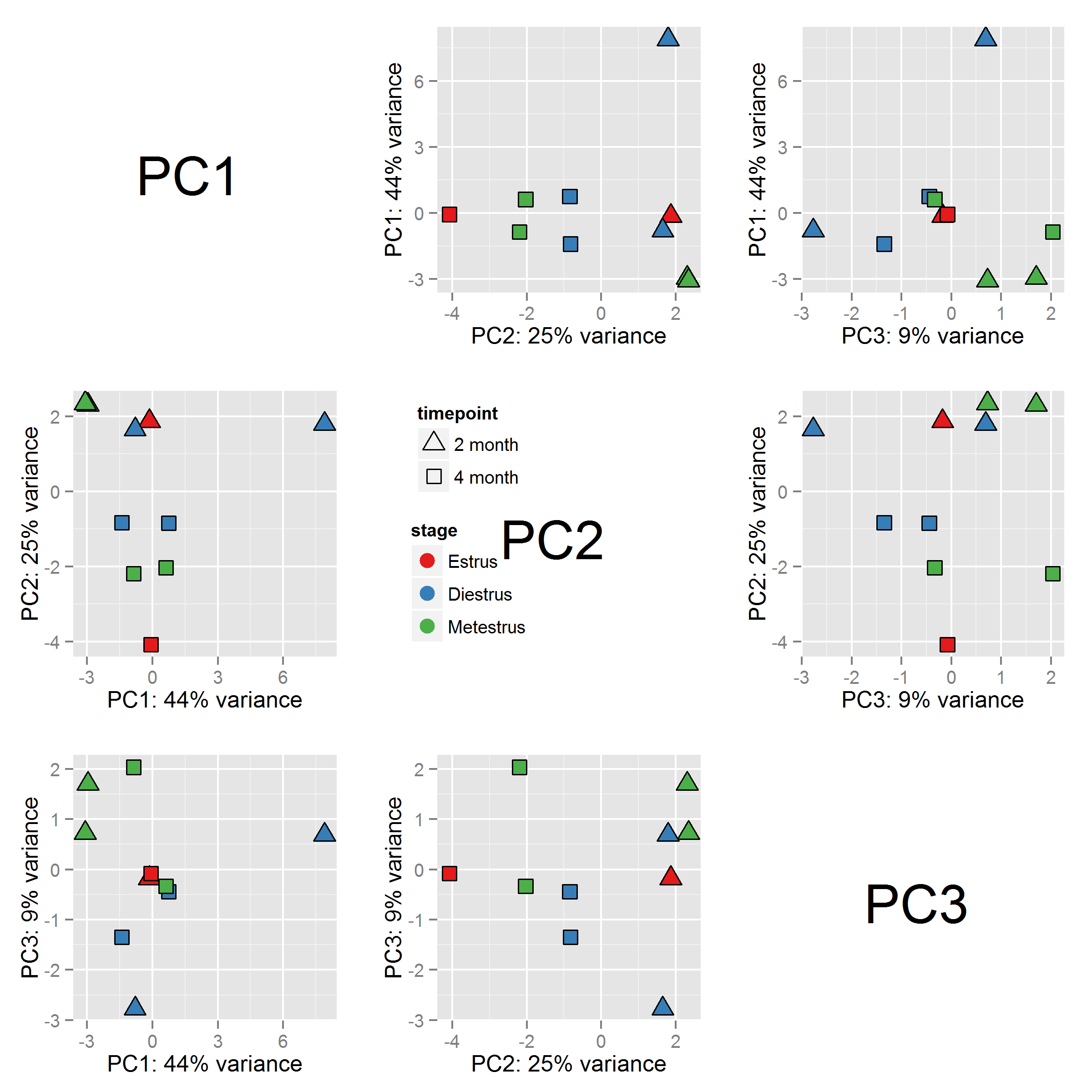

Supplement: Additional file 5: Figure S1. — Principal Component Analysis of only female samples. File contains figure of principal component analysis of 2 and 4 month old female samples to investigate variance in gene expression associated with differences in estrous stage. (PNG 96 kb) [file 12864_2017_3608_MOESM5_ESM.png]
